# Methods to Increase the Metabolic Stability of ^18^F-Radiotracers

**DOI:** 10.3390/molecules200916186

**Published:** 2015-09-03

**Authors:** Manuela Kuchar, Constantin Mamat

**Affiliations:** Helmholtz-Zentrum Dresden-Rossendorf, Institut für Radiopharmazeutische Krebsforschung, Bautzner Landstraße 400, Dresden D-01328, Germany; E-Mail: m.kuchar@hzdr.de

**Keywords:** fluorine-18, metabolism, stability, deuterium

## Abstract

The majority of pharmaceuticals and other organic compounds incorporating radiotracers that are considered foreign to the body undergo metabolic changes *in vivo*. Metabolic degradation of these drugs is commonly caused by a system of enzymes of low substrate specificity requirement, which is present mainly in the liver, but drug metabolism may also take place in the kidneys or other organs. Thus, radiotracers and all other pharmaceuticals are faced with enormous challenges to maintain their stability *in vivo* highlighting the importance of their structure. Often in practice, such biologically active molecules exhibit these properties *in vitro*, but fail during *in vivo* studies due to obtaining an increased metabolism within minutes. Many pharmacologically and biologically interesting compounds never see application due to their lack of stability. One of the most important issues of radiotracers development based on fluorine-18 is the stability *in vitro* and *in vivo*. Sometimes, the metabolism of ^18^F-radiotracers goes along with the cleavage of the C-F bond and with the rejection of [^18^F]fluoride mostly combined with high background and accumulation in the skeleton. This review deals with the impact of radiodefluorination and with approaches to stabilize the C-F bond to avoid the cleavage between fluorine and carbon.

## 1. Introduction

Positron emission tomography (PET) and the combined techniques PET/MRT and PET/CT are outstanding imaging instruments and allow for the quantification and localization of physiological as well as pathophysiological processes *in vivo*, which were analyzed by tracing the appropriate biochemical fundamentals [1]. The basics of PET originate in the coincidental detection of annihilation photons emitted 180° apart, which originate from the radiotracer emitting positron, which again collides with electrons in the surrounding tissue. Measurement and quantification of the tracer distribution were obtained noninvasively in living organisms [2]. Fluorine-18 is an ideal radionuclide due to its favorable nuclear decay properties. It has a half-life of 109.8 min, which provides sufficient time to radiolabel the molecule of interest and localize it *in vivo*. Additionally, it emits a positron of low kinetic energy, which only travels a short range in tissue leading to high image resolution. However, tracers for PET imaging are always restricted by the kind of molecules that researchers can prepare and label. A summary of commonly used PET radionuclides is found in Table 1.

**Table 1:** Most commonly used PET radionuclides with their radiochemical details [3].

Nuclide	*t*_½_ (min)	Production Route	Average Range in H_2_O (mm)	E_av._ (β^+^) (keV)
^11^C	20.4	^14^N(p,α)^11^C	1	385
^13^N	10	^16^O(p,α)^13^N	1.5	491
^15^O	2	^15^N(d,n)^15^O	2.7	735
^18^F	109.8	^20^Ne(d,α)^18^F ^18^O(p,n)^18^F	0.3	242
^68^Ga	67.6	^68^Ge-^68^Ga generator	3.7	740
^124^I	250.6	^124^Te(p,n)^124^I	3	188

Fluorine-18 is a unique radionuclide for PET imaging. In contrast to other β^+^ emitting organic radionuclides like ^11^C, ^13^N, and ^15^O, which are inclined to isotopic labeling, fluorine-18 is most commonly incorporated leading to an alteration of the original compound [4,5]. Due to the absence of fluorine in nearly all naturally occurring biomolecules [6], radiolabeling is often accomplished by a formal replacement of a proton or an OH group with ^18^F (isosteric and isopolar) which is known as bioisosteric labeling (Table 2) [7]. However, in medicinal chemistry, the role of fluorine in drug design and development is expanding rapidly and a wide variety of small compounds/drugs were developed in the past with pharmacological relevance still having one or more fluorine atoms inside [8]. These molecules can serve as brilliant lead structures for ^18^F-radiotracers. The other variant deals with the connection of small ^18^F-building blocks or ^18^F-prosthetic groups like [^18^F]SFB or [^18^F]FBAM [9], but this is mostly used with biomacromolecules like peptides, proteins, or antibodies. Both methods come along with changes of biological and/or pharmacological properties of the tracer molecule compared to the original compound. In general, smaller molecules exhibit a larger change in their properties by the introduction of a radionuclide to the considered molecule.

### 1.1. Nature of the C-F Bond

The similarity in size of fluorine (147 pm), hydrogen (120 pm) and oxygen (152 pm) makes fluorine-18 an appropriate candidate for the preparation of radiotracers, due to its longer half-life time compared to ^11^C, ^13^N or ^15^O (Table 1) [10]. The substitution of single hydrogen or a hydroxyl group by fluorine induces only a slight steric perturbation [11]. The similarity of the C-F to the C-O bond length (Table 2) and the similar electronic properties like the induced dipole due to the inductive effect allows the isoelectronic replacement of an OH group by fluorine [12]. However, fluorine is only a (poor) hydrogen bond acceptor, while an OH group is both a hydrogen donor and an acceptor.

**Table 2:** Van der Waals radii [13], electronegativity and aliphatic C-X bond lengths of selected atoms.

Element X	Van der Waals Radius (pm)	Electronegativity (Pauling Scale)	Bond Length of C-X (pm)
H	120	2.1	109
C	170	2.5	154
O	152	3.5	143
F	147	4.0	135

Of all the atoms, the fluorine atom possesses the highest electronegativity; therefore, biological aspects have to be considered and can be of advantage in pharmaceutical as well as in radiotracer design. A favorable feature of fluorine is the strong but highly polarized σ bond to carbon [11]; this should make the fluorine a perfect leaving group in case of nucleophilic displacement reactions. However, the fluorine unexpectedly does not show good donor ability despite the high polarization of the C-F bond. This fact can be explained by the strong interaction of the partially positively charged carbon (residue) and the partially negatively charged fluorine which results in the strongest known σ bond in organic chemistry. Additionally, the highest bond dissociation energy (BDE) of approx. 441.3 kJ/mol is found for an aliphatic C-F bond compared to other carbon single bonds [14].

**Scheme 1:**
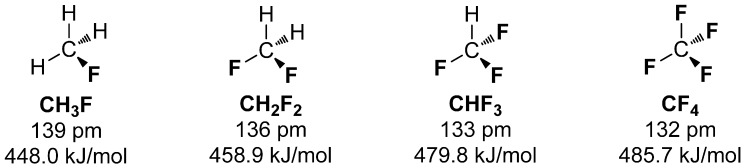
Comparison of fluoromethanes with increasing number of bound fluorine and their associated bond length and bond dissociation energy.

Interestingly, the average BDE differs with the number of covalently bound fluorine. More fluorine atoms bound to carbon increases the BDE and diminishes the C-F bond length [15]. The series of fluoromethane compounds in Scheme 1 demonstrate this trend, which can be explained by each of the C-F bonds pulling p-orbital electron density from the sp^3^ carbon to the low lying sp^2^ orbitals of fluorine (Bent’s rule [16]), making the carbon more sp^2^ in character [17].

Particular attention has to be made for fluorine in the benzylic position. It was found that the bond enthalpies (DH_298_) are lower for benzyl fluorides (413.2 kJ/mol) compared to aliphatic compounds (439.2 kJ/mol for methyl fluoride) or aromatic fluorine derivatives (472.7 kJ/mol for fluorobenzene) [11]. Furthermore, the C-F bond length is also extended (138 pm compared to 135 pm average) [18]. The remaining substituents on the aromatic ring also have crucial influence on the stability of the benzylic fluorine especially concerning steric and inductive effects [19]. The introduction of [^18^F]fluoride into the benzylic position of precursors occurs as previously described for aliphatic compounds, under milder reaction conditions in most of the cases due to the comparatively higher reactivity of the benzylic position. In this regard, the metabolic stability of benzyl [^18^F]fluorides is also decreased [20]. Additionally, similar observations were made for allyl fluorides [21].

Aromatic C_Ar_(sp^2^)-F bonds are stronger than aliphatic C(sp^3^)-F bonds [22] resulting in their bond lengths being shorter: 140 pm (aliphatic) *vs.* 136 pm (aromatic) [23]. This finding can be explained by the high polarization of the σ-C-F bond, the possible delocalization of the (partial) positive charge of the carbon in the aromatic moiety, and that fluorine possibly acts as a π-electron donor [24,25], which strengthens the C-F bond additionally. Thus, the ^18^F radiolabeling of aromatic systems should be favored over aliphatic systems.

Bonding of fluorine to a sp-hybridized carbon is also possible, but this results in a highly reactive species due to the repulsion of the free electron pairs of the fluorine and the π-electron system of the triple bond [17]. This trend strongly follows Bent’s rule, which states that the s-character of an atom concentrates in orbitals directed toward electropositive substituents [16]. No ^18^F-radiotracer with a direct connection of ^18^F to a triple bond has been developed to date.

To sum up, to produce a stabilized C-F bond, the most important criterion is the hybridization of the carbon. Moreover, inductive and steric effects of further substituents and organic residues influence the (metabolic) stability of the C-F bond as well.

### 1.2. Possibilities to Introduce Fluorine-18—Short Overview

The radionuclide ^18^F is produced by a cyclotron using the nuclear reactions shown in Table 1. Once the radionuclide is produced, it must quickly be incorporated in the molecule of interest. Normally, the introduction of ^18^F into aliphatic molecules (sp^3^-hybridized carbon) is accomplished using no-carrier-added (n.c.a.) [^18^F]fluoride and a precursor with a good leaving group (Br, I, OMs, OTs, ONs, OTf, NR_3_^+^) in a S_N_2 reaction. This method has the advantage of preparing radiotracers with high specific activity (A_S_). A challenging aspect of this labeling procedure is to eliminate traces of water to remove the hydration shell around the fluoride. Polar organic solvents (ACN, DMF, DMSO) were used with a cryptand (Krypofix K2.2.2.) to function as a phase transfer catalyst and to further separate the charge of the cation and fluoride (producing what is called naked fluoride) [26].

Introduction of fluorine-18 into aromatic systems can be performed by several reaction pathways. The classical Balz-Schiemann reaction is only rarely used for this purpose [27]. Commonly, two ways are applied: the nucleophilic aromatic substitution (S_N_Ar) and the electrophilic aromatic substitution (S_E_Ar). The major drawback when using the first variant is the necessity to activate the respective aromatic precursor with electron withdrawing groups (CN, halogens, NO_2_, C=O) as well as good leaving groups. An isotopic exchange of ^19^F by ^18^F is also possible, but this results in a low A_S_. This is reasoned by the disability to separate the ^19^F-precursor from the ^18^F-radiotracer. This is because the A_S_ is always influenced by the applied amount of the ^19^F-compound. Other appropriate leaving groups for the nucleophilic aromatic displacement are halogens, NO_2_ or Me_3_N^+^. Newer developments are based on iodonium [28] or sulfonium salts [29,30] as precursors and can be used for non-activated aromatic systems as well [31,32].

Using S_E_Ar, [^18^F]F_2_ was applied consisting of both ^18^F and ^19^F (carrier added, c.a.), thus, the labeling will proceed in an “electrophilic” manner. As a consequence, a minimum of 50% of the elemental fluorine is ^19^F and therefore not β^+^-decaying. This pathway leads to a reduced A_S_ of the radiotracers due to the incorporation of ^19^F. Usually, stannylated precursors, in which the carbon has the partial negative charge, are required for the labeling with [^18^F]F_2_ [33].

## 2. Radiodefluorination

Today’s arsenal of radiotracers comprises more and more complex molecules ranging from small organic and pharmacologically active derivatives such as carbohydrates, amino acids or steroids to high molecular weight compounds like peptides, proteins or oligonucleotides. The development of new radiotracers for molecular imaging has to address important questions on target selection and radiobiological validation. These special requirements are encountered in radiotracer synthesis such as choice of the appropriate radionuclide and suitable labeling position. In this regard, a radiotracer has to meet different criteria to be delivered to the target area of interest such as an adequate lipophilicity, high selectivity to the biological target and a high metabolic stability *in vivo* [34]. Hence, special attention should be paid to implement fast and highly selective labeling reactions for radiotracers which tolerate other functional groups. One of the most important aspects in the design of new radiopharmaceuticals is the development of metabolically stable tracers to meet the desired requirements and characteristics as mentioned above [35]. The radiolytic decomposition of ^18^F-radiotracers is also an important issue, especially during isolation and formulation of the tracer. This drawback can be avoided using additives like anti-oxidant stabilizers [36].

Drug metabolism, also known as xenobiotic metabolism, involves the biochemical modification of substances (pharmaceuticals, drugs, poisons, radiotracers). Drugs often are foreign compounds to the organism’s normal biochemistry. This metabolism usually occurs through specialized enzymatic systems by living organisms. Because of this mechanism, lipophilic substances are often converted into more readily hydrophilic derivatives, which are then excreted. The rate of metabolism determines the duration and efficacy of a drug, also known as the biological half-life [37]. In the case of radiopharmaceuticals, the physical half-life of the appendant radionuclide influences this mechanism supplementary.

The reactions in these biochemical pathways are of particular interest in medicine as part of drug metabolism and as a factor contributing to multidrug resistance in infectious diseases, cancer chemotherapy or radiopharmacy. The speed of the homing process of a radioactive drug has to be relatively fast compared to the biological and physical half live of the drug to be able to obtain good signal to background ratio.

Drug metabolism in general is divided into three phases. In phase I, enzymes such as cytochrome P450 oxidase (oxidative metabolism: CYP, FMO, MAO, Mo-CO, aldehyde oxidase, peroxidases, xanthine oxidase; hydrolytic metabolism: esterase, amidases, epoxide hydrolases) introduce reactive or polar groups into the xenobiotics. Afterwards, these modified compounds are conjugated to yield more polar compounds in phase II reactions. These reactions are catalyzed by transferase enzymes (UGT, ST, NAT, GST, MT) [38]. Finally, in phase III, the conjugated xenobiotics may be further processed, before being recognized by efflux transporters and eliminated from the cells. Radiotracers follow this method of degradation as well with one large difference; they are administered in concentrations, which are significantly lower than “normal” pharmaceuticals.

**Scheme 2:**
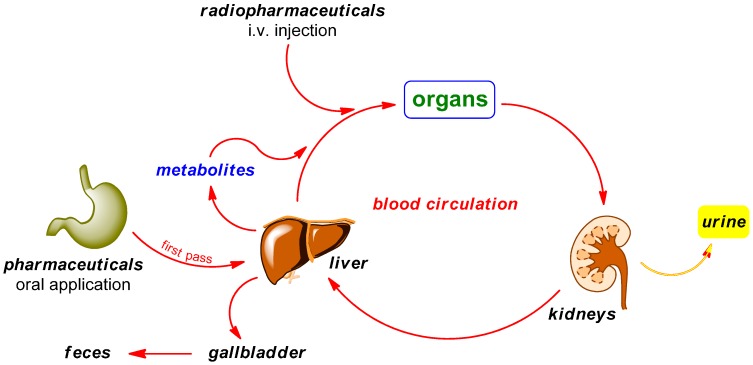
Possible pathways of radiopharmaceuticals in contrast to pharmaceuticals in the body.

PET radiotracers are typically injected intravenously in contrast to the orally administered “normal” pharmaceuticals. While circulating in the blood and tissues prior to localizing at the target site, a portion of the drug may be metabolized. The major organs involved in this metabolism process are the kidneys and liver. The biotransformation may happen within minutes of administration and the resulting radiometabolites are generally less lipophilic than the original radiotracer. Possible metabolic degradation pathways are illustrated in Scheme 2. Radiodefluorination is known to be a phase I reaction occurring primarily through the action of cytochrome P450 2E1 (CYP2E1) isozyme in liver microsomes [39,40,41].

### Mechanisms of Radiodefluorination

Radiotracers, independent on their corresponding radionuclide, that resist extensive metabolism *in vivo* over the period of time of a PET scanning session are seldom [42]. Metabolic paths that derivatize rather than disintegrate the respective tracer into small, more polar fragments, can produce unpleasant radiometabolites. Alongside the formation of more polar radiometabolites, the cleavage of [^18^F]fluoride from the tracer, also known as radiodefluorination, is a major way for several ^18^F-radiotracers to degrade despite the high strength of the C-F bond [43]. Afterwards, [^18^F]fluoride as the main metabolite binds primarily to bone and skull. Especially while imaging the central nervous system, [^18^F]fluoride binding to the skull is problematic [42].

**Scheme 3:**
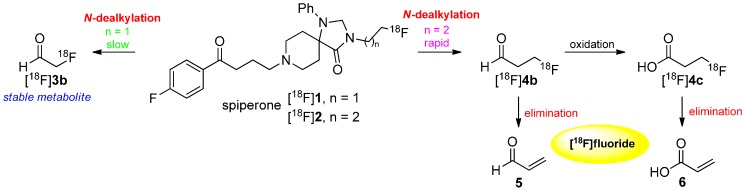
Mechanism of degradation of [^18^F]**1** and [^18^F]**2** leading to *N*-dealkylation of the radiotracer.

There are several metabolic pathways discussed and proposed for the degradation of [^18^F]fluoroalkyl chains. Two major factors affect the method of degradation: the location of the fluoroalkyl chain in the molecules and their length. In 1988, Welch and co-workers showed the difference in the metabolism between *N*-[^18^F]fluoroethylated and *N*-[^18^F]fluoropropylated spiperones [^18^F]**1** and [^18^F]**2** [44]. They proposed a metabolization by *N*-dealkylation followed by oxidation to the respective [^18^F]fluoroaldehydes [^18^F]**3b** and [^18^F]**4b**. As a result, 2-[^18^F]fluoroacetaldehyde ([^18^F]**3b**) is a stable lipophilic metabolite whereas 3-[^18^F]fluoropropanal ([^18^F]**4b**) is unstable towards elimination of [^18^F]fluoride (retro Michael addition). Further oxidation led to 3-[^18^F]fluoropropionate ([^18^F]**4c**), which eliminated subsequently to [^18^F]fluoride, too. In general, this kind of metabolism occurs when [^18/19^F]fluoroalkyl chains are bound to heteroatoms like oxygen, nitrogen or sulfur. Both pathways are shown in Scheme 3.

**Scheme 4:**
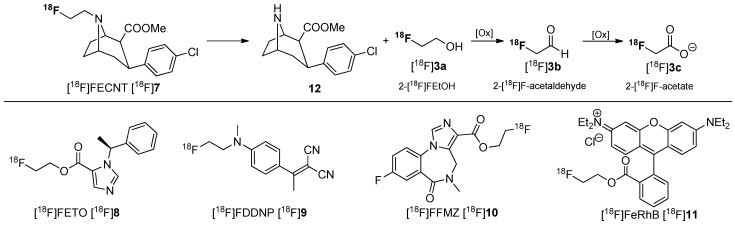
Selected [^18^F]fluoroethylated tracers and assumed metabolic pathway for degradation of [^18^F]FECNT [^18^F]**7**.

In many cases, 2-[^18^F]fluoroethanol [^18^F]**3a** or its metabolites 2-[^18^F]fluoroacetaldehyde [^18^F]**3b** and 2-[^18^F]fluoroacetate [^18^F]**3c** have been observed during metabolic degradation of several PET tracers containing a 2-[^18^F]fluoroethyl group such as [^18^F]FECNT [^18^F]**7** [45], [^18^F]FETO [^18^F]**8** [46], [^18^F]FDDNP [^18^F]**9** [47], [^18^F]FFMZ [^18^F]**10** [48], and [^18^F]FERhB [^18^F]**11** [49]. The metabolites (presumably 2-[^18^F]fluoroacetaldehyde [^18^F]**3b** and 2-[^18^F]fluoroacetate [^18^F]**3c**) from *N*-defluoroethylation of [^18^F]FECNT [^18^F]**7** and [^18^F]FDDNP [^18^F]**9** have been shown to distribute evenly in the brain, confounding data analysis [46,47]. The metabolic behavior of [^18^F]FECNT [^18^F]**7** is shown in Scheme 4.

The behavior of 2-[^18^F]fluoroethanol ([^18^F]**3a**) and 3-[^18^F]fluoropropanol ([^18^F]**4a**) as possible radiometabolites was further investigated during the process of radiodefluorination [50]. The basis of these experiments was the assumption that fluoroalkyl ethers and ester were cleaved to give both aforementioned radiometabolites. Further, it was suggested that 2-[^18^F]fluoroethanol ([^18^F]**3a**) is converted to 2-[^18^F]fluoroacetaldehyde ([^18^F]**3b**), which is then metabolized to 2-[^18^F]fluoroacetate ([^18^F]**3c**). After formation of 2-[^18^F]fluoroacetyl-CoA, it remains trapped inside the cell [51]. No activity was found in the bone, but it was stated that 2-[^18^F]fluoroethanol ([^18^F]**3a**) behaves like H_2_[^15^O]O.

In contrast, when investigating 3-[^18^F]fluoropropanol ([^18^F]**4a**) *in vivo* as a potential radiometabolite of 3-[^18^F]fluoropropylated PET tracers a rapid accumulation in the skeleton was observed. This can be explained by free [^18^F]fluoride being generated from 3-[^18^F]fluoropropionaldehyde ([^18^F]**4b**) or 2-[^18^F]fluoropropionate ([^18^F]**4c**). Both metabolites leading to further β-elimination under release of [^18^F]fluoride (vide supra).

**Scheme 5:**
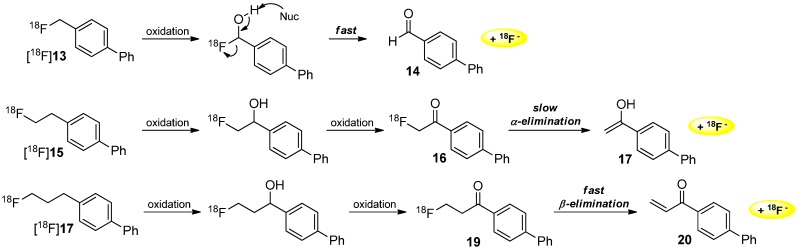
Different metabolic behavior of [^18^F]fluoroalkylated aromatic compounds.

Furthermore, Lee and co-workers demonstrated the radiodefluorination of [^18^F]fluoroalkyl groups bound to an aromatic system [52]. Several biphenyl derivatives ([^18^F]**13**, [^18^F]**15**, [^18^F]**17**) and their degradation behavior were investigated. In all evaluated reactions, the first step consists of the oxidation of the carbon next to the aromatic ring. In the case of the fluoromethyl group of [^18^F]**13**, a fast elimination step followed to give the respective aldehyde and [^18^F]fluoride. In the case of the fluoroethyl residue of [^18^F]**15**, a slow α-elimination occurred to give [^18^F]fluoride and the remaining enol (ketone). In the third case, β-elimination took place after oxidation of [^18^F]**17** to an α,β-unsaturated system **20** and elimination of [^18^F]fluoride. The different metabolic degradation of these compounds is investigated (Scheme 5).

**Scheme 6:**
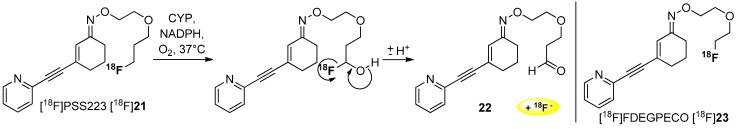
Release of [^18^F]fluoride from [^18^F]PSS223 [^18^F]**21** during degradation with cytochrome P450 (CYP).

Schibli and co-workers gave an alternative explanation for the mechanism of radiodefluorination. They assumed an oxidation of the carbon next to the [^18^F]fluorine of tracer [^18^F]PSS223 [^18^F]**21** involved by cytochrome P450 enzyme (CYP) leading to a separation of [^18^F]fluoride (Scheme 6). Experiments performed *in vivo* showed an accumulation of [^18^F]fluoride in the bone [53] as reported above.

Stability determinations with [^18^F]PSS223 [^18^F]**21** using rat and human liver microsomal enzymes were executed and pointed out two more polar radiometabolites as demonstrated by radio-UPLC measurements. The degradation process is shown to be NADPH-dependent, which implied the involvement of oxidoreductases. Amongst others, the fluorine-containing carbon atom was oxygenated leading to the release of [^18^F]fluoride.

The difference in the *in vivo* behavior between [^18^F]PSS223 [^18^F]**21** and [^18^F]FDEGPECO [^18^F]**23** could be explained by the β-heteroatom effect [54,55], by which primary aliphatic bound [^18^F]fluorine in β-position to heteroatoms (e.g., ROCH_2_CH_2_[^18^F]F) is found to be metabolized at a slower rate. This rationale supports the absence of defluorination for [^18^F]FDEGPECO [^18^F]**23** containing only a [^18^F]fluoroethyl group.

## 3. Methods to Avoid Radiodefluorination

The probably best alternative to avoid radiodefluorination consists of the direct connection of fluorine-18 to a phenyl moiety instead of aliphatic residues wherever applicable [56]. This is consistent with the higher stability of a C_Ar_-F bond compared to a C(sp^3^)-F bond as previously described. The fluoroaryl groups are stable to metabolism and do not lead to a considerable radiodefluorination. Otherwise, the [^18^F]fluoroalkyl moiety has to be modified to reduce or avoid rapid metabolic degradation by the following methods.

### Deuteration in Direct Neighborhood of Fluorine-18

A fundamental approach in medical chemistry is the application of deuterium to increase the stability of active pharmaceutical ingredients [57], which is useable to raise the metabolic stability of ^18^F-radiotracers by means of the deuterium-proton exchange at carbon atoms close to the ^18^F-atom. This procedure can sometimes suppress but not completely prevent the process of radiodefluorination.

**Scheme 7:**
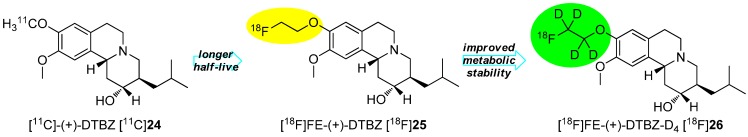
Enhancement of the half-life as well as of the metabolic stability of DTBZ **24**.

The method of action of this effect is explained by the kinetic isotope effect that reduces the rate of metabolic degradation. The deuterium is not only twice as heavy as hydrogen, but also the zero-point energy is significantly lower than the energy of hydrogen. Due to these differences, the activation energy of the C-D-bond in chemical or biochemical reactions is significantly higher than for C-H-bond. Therefore, reactions on the C-D-bond will proceed considerably slower than the same reactions with a C-H-bond at the same position. In general, the cleavage rate of a C-H bond is 6.7 times faster compared to a C-D bond at 25 °C and it is postulated that the break of the C-H bond is the rate-determining step in this kind of defluorination [58,59]. A successful example of the stabilization by means of deuteration consists of the preparation of [^18^F]FE-DTBZ-D_4_ [^18^F]**26**, which is pointed out in Scheme 7.

[^11^C]-(+)-DTBZ [^11^C]**24** was initially used to studied dementia and Parkinson in the clinic [60,61,62]. Further improvements were necessary including the change of the radionuclide to elongate the half-live, which led to the development of [^18^F]FE-(+)-DTBZ [^18^F]**25** [63,64]. Successful *in vitro* studies with this ^18^F-tracer were accomplished followed by *in vivo* studies showing a high accumulation of radioactivity in joints and bones. To improve the metabolic stability, [^18^F]FE-(+)-DTBZ-D_4_ [^18^F]**26** was developed and showed enhanced properties. The main improvement resulted in the considerably reduced bone uptake when comparing both tracers. The defluorination rate (*k*_defluorination_) was determined for both tracers to be 0.012 for [^18^F]**25** and 0.0016 for [^18^F]**26** resulting in an elongated plasma-t_1/2_ from 46.2 min to 438.7 min [65].

The example in Scheme 7 exhibits the introduction of a deuterated [^18^F]fluoroalkyl residue via a building block strategy using a fluorine-18 containing deuterated building block. The second general method consists of the gradual introduction of the deuterium followed by ^18^F-labeling as the last step. In this case, the precursor already possesses the deuterium.

**Scheme 8:**
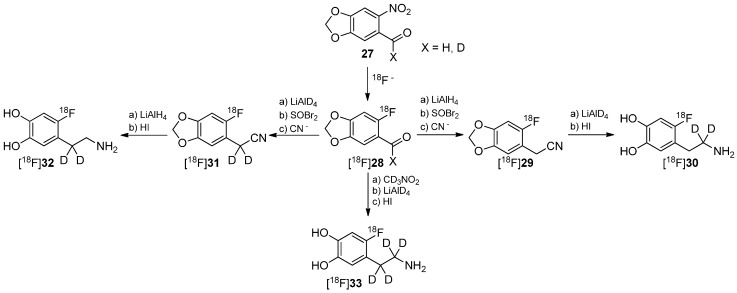
Synthesis of different regioselectively deuterated [^18^F]fluorodopamine derivatives.

One of the first reports regarding the introduction of deuterium into precursors to prepare ^18^F-radiotracers was presented by Ding, Fowler and Wolf in 1993 [66]. They introduced deuterium in different positions of the alkyl chain of 6-[^18^F]fluorodopamine (6-[^18^F]FDA) regioselectively to execute mechanistic studies regarding the degradation of these derivatives ([^18^F]**30**, [^18^F]**32**, [^18^F]**33**) by monoamine oxidase B (MAO B) and dopamine β-hydrolase (DBH) via PET. The reaction path to precursors and resulting radiotracers is shown in Scheme 8.

It has recently been shown that MAO B and DBH stereoselectively remove only the pro-R hydrogen of the non-deuterated compounds [67,68,69,70]. Both dopamine compounds [^18^F]**30** and [^18^F]**32** with two deuterium atoms on one carbon were prepared to further verify this finding. Such specifically deuterated derivatives are therefore the most appropriate candidates for unambiguously assessing the contribution of metabolism by MAO and DBH on the kinetics of 6-[^18^F]fluorodopamine.

**Scheme 9:**
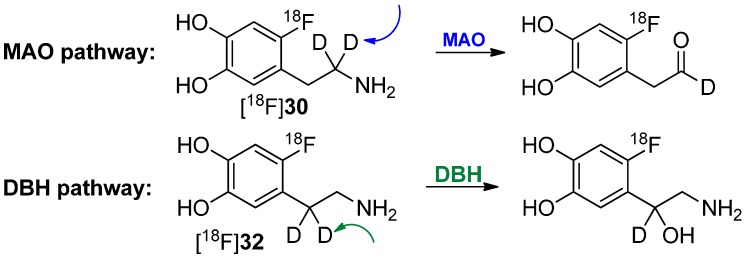
Metabolic conversion of [^18^F]**30** and [^18^F]**32** by DBH and MAO, respectively.

In a following paper, it was shown that [^18^F]**30** has a reduced rate of clearance, consistent with MAO-catalyzed cleavage of the α-C-D bond, whereas [^18^F]**32** showed no change, indicating that cleavage of the β-C-D bond (DBH) is not rate limiting [71]. Both pathways of degradation are shown in Scheme 9. Furthermore, the rate of metabolism was also significantly reduced by pretreatment with pargyline (MAO inhibitor).

**Scheme 10:**
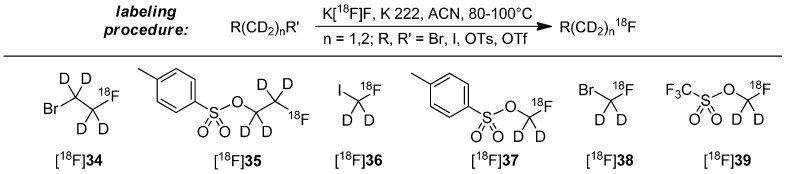
General labeling procedure to create the deuterated building blocks and known building blocks [^18^F]**34**–[^18^F]**39**.

The most often applied approach of the stabilization with deuterium consists of the use of deuterated [^18^F]fluoroalkyl building blocks. For preparation, dihalogens, disulfonates or derivatives with mixed functions were used as starting material with deuterated methylene (-CD_2_-) or ethylene (-CD_2_CD_2_-) groups. The introduction of ^18^F follows standard labeling conditions (K222, anhydrous acetonitrile, 80–100 °C, 15–30 min). Examples for building blocks and most common labeling conditions to prepare these building blocks are shown in Scheme 10 [55,65,72,73,74,75,76,77,78,79,80].

The subsequent labeling procedure with the above mentioned building blocks [^18^F]**34**–[^18^F]**39** represents a nucleophilic displacement at the carbon of the building block. Normally, sulfonate leaving groups are superior to halogens, but Schou and co-workers demonstrated that the type of the leaving group has only a small influence on the radiochemical yield (RCY) of the resulting radiotracer [72]. Mostly, the final alkylation reaction of building block with precursor proceeds rapidly (approx. 5 min reaction time) [65,72].

**Scheme 11:**
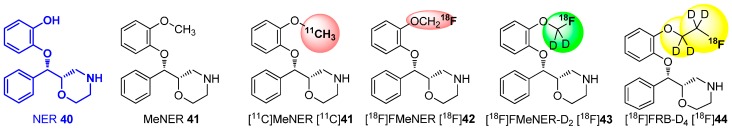
Carbon-11, fluorine-18 and deuterated derivatives of NER **40** to increase metabolic stability.

Amongst others, this building block strategy was extensively investigated for MeNER **41** (Scheme 11), which was identified as high-affinity ligand (IC_50_: 2.5 nM *in vitro*) for the norepinephrine transporter (NET). Imaging of the NET moved into focus of research to investigate several neuropsychiatric and neurodegenerative disorders. The first successful PET images were obtained with carbon-11 labeled derivative [^11^C]MeNER [^11^C]**41**, which was synthesized by the use of NER **40** as precursor and radiolabeled with [^11^C]MeOTf. Unfortunately, the binding of this MeNER derivate [^11^C]**41** to the NET proceeded within a range of about 90 min *in vivo*, which was too long for a carbon-11 labeled tracer (*t*_½_ = 20.4 min) [81]. This, led to the development of [^18^F]FMeNER [^18^F]**42**, an improved tracer with fluorine-18 (*t*_½_ = 109.77 min) on the methyl group. [^18^F]**42** still bound to the receptor with high affinity, while providing a sufficient half-life for imaging. This tracer was synthesized from the same precursor using bromo[^18^F]fluoromethane and [^18^F]fluoromethyl triflate with similar results. In contrast, the signal-to-background ratio and the bone uptake was increased compared to PET images from [^11^C]MeNER [^11^C]**41**. Fortunately, this effect was nearly completely suppressed by the use of the deuterated derivative [^18^F]FMeNER-D_2_ [^18^F]**43**, which shows the impact of the isotope effect for the development of radiotracers [72].

This successful procedure was also applied for the preparation of [^18^F]FRB, the ethoxy derivative of MeNER **41** based on Reboxetine (IC_50(NET)_: 8.23 nM). For this purpose, the precursor NER **40** was successful labeled with [^18^F]fluoroethyl bromide and [^18^F]fluoroethyl bromide-D_4_ ([^18^F]**34**), to give [^18^F]FRB and [^18^F]FRB-D_4_ [^18^F]**44**, respectively. Due to the better pharmacological properties of [^18^F]FMeNER-D_2_ [^18^F]**43** compared to [^18^F]FRB-D_4_ [^18^F]**44**, a fully automated synthesis was developed for [^18^F]FMeNER-D_2_ [^18^F]**43** in 2013 [80].

**Scheme 12:**
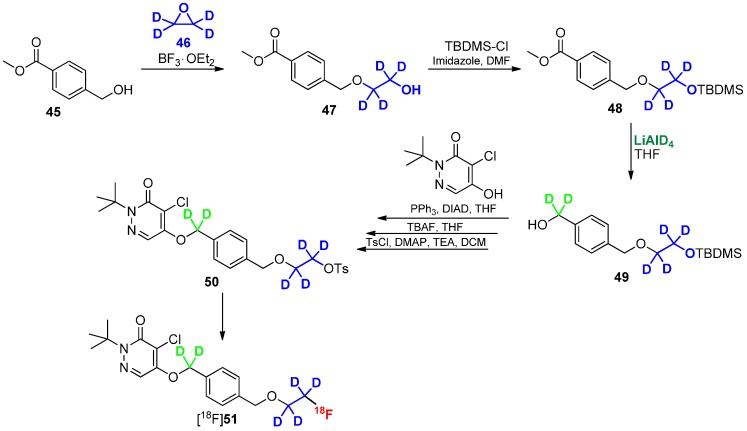
Direct approach to introduce deuterium and fluorine-18 into radiotracer [^18^F]**51**.

Another promising approach to use deuterated building blocks was shown by Casebier and colleagues [82]. In contrast to the previously discussed approaches, the deuterium containing residue was directly connected to the precursor molecule prior to radiolabeling to avoid a two-step-synthesis of radiotracer. The interesting task of this work was the use of fully deuterated ethylene oxide as building block (Scheme 12), which was introduced via ring-opening reaction. The next steps required the protection of the OH group with TBDMS-Cl followed by reduction of the methyl ester with LiAlD_4_. The obtained deuterated methylene group is mandatory for a further stabilization of the tracer in terms of metabolic degradation. Upon completion of the basic structure of the molecule, the hydroxyl group was selectively deprotected using TBAF and functionalized with *p*-tosylchloride for labeling with fluorine-18.

**Scheme 13:**
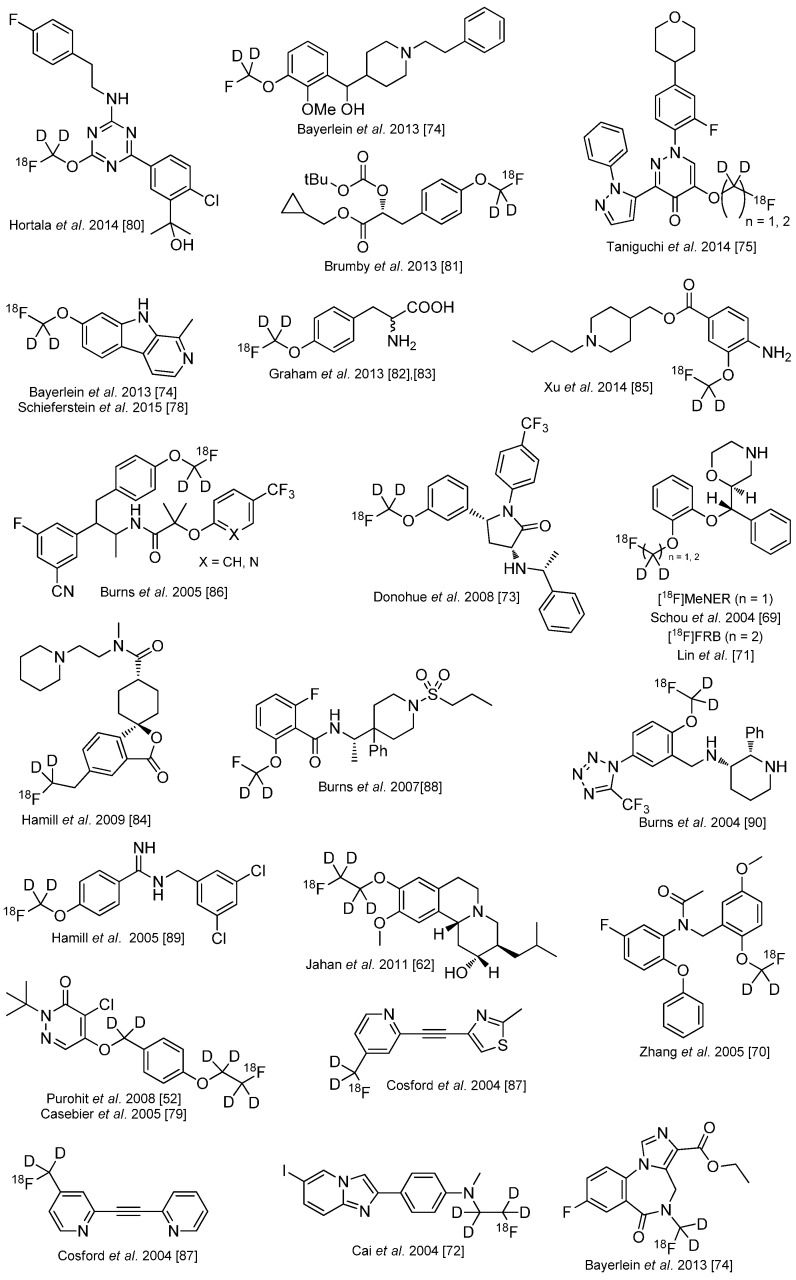
Overview over ^18^F-radiotracers stabilized with deuterium in direct neighborhood to ^18^F.

Several other ^18^F-tracers are known which are stabilized with deuterium. Selected examples are given in the following overview in Scheme 13 [83,84,85,86,87,88,89,90,91,92,93]. As stated before, the radiodefluorination process cannot fully be avoided, but it can be delayed considerably.

## 4. Deuteration on other Parts of the Molecule to Avoid Degradation

**Scheme 14:**
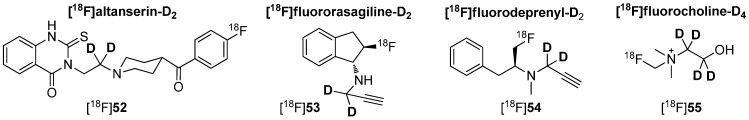
Examples for radiotracers deuterated on other parts of the molecule.

As already mentioned, hydrogen–deuterium exchange will not only be adopted in direct proximity to fluorine-18 to avoid radiodefluorination or other metabolic degradation. A similar effect could be achieved by the use of deuterium connected to endangered areas in the tracer molecule, which are prone to metabolic degradation. Such an additional stabilization was already shown for compound [^18^F]**51** by Casebier and colleagues in Scheme 12 [82]. Furthermore, the application of deuterium leads occasionally to another metabolic pathways as it was figured out by Leyton, Smith and co-workers [94,95]. Some examples for ^18^F-radiotracers deuterated on other parts of the molecule are shown in Scheme 14.

[^18^F]Fluororasagiline-D_2_ ([^18^F]**53**) and [^18^F]fluorodeprenyl-D_2_ ([^18^F]**54**) (Scheme 14) are two examples of a successful enhancement of the metabolic stability using deuterium. Both tracers are known to be inhibitors of monoamnooxidase (MAO) and were applied for detection of psychiatric and neurological disorders such as depression, Alzheimer, and Parkinson diseases [96]. Although both lead structures (rasagiline and L-deprenyl) contain a propargyl group, there are significant differences in their metabolic products [97]. Inhibition experiments *in vitro* pointed out a high selectivity of MAO-B compared to MAO-A for both above mentioned radiotracers as well as for their non-deuterated derivatives. Moreover, it was found that the alkynyl chain of these molecules was cleaved under *in vivo* conditions. Thus, deuterium was selectively introduced into this residue to stabilize these molecules.

Comparing the deuterated and non-deuterated tracers in terms of their radiopharmacological behavior *in vivo*, [^18^F]fluorodeprenyl showed a fast and irreversible binding to the enzyme limited by blood flow rather than by the MAO-B enzyme concentration, whereas [^18^F]fluororasagiline expressed continuous increase of the radioactivity in the brain indicating a blood–brain barrier penetrating radiometabolite. In contrast, [^18^F]fluororasagiline-D_2_ ([^18^F]**53**) and [^18^F]fluorodeprenyl-D_2_ ([^18^F]**54**) exhibited fast clearance from the brain and less accumulation in cortical and sub-cortical regions. Furthermore, both deuterated analogues were more stable in monkey plasma compared to their non-deuterated analogues [98]; metabolic degradation was almost completely reduced. Thus, the deuterated tracers seemed to be more suitable for an application over the non-deuterated derivatives.

**Scheme 15:**
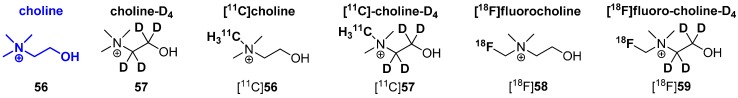
Several choline derivatives with and without deuterium labeled with carbon-11 or fluorine-18.

Another interesting example is the metabolic behavior of radiolabeled cholines. Known radiolabeled derivatives are exemplified in Scheme 15. In general, two main metabolic pathways are known for choline derivatives. The first pathway is based on the phosphorylation of choline via choline kinase (E.C. 2.7.1.32) to phosphocholine which is further transformed to phosphatidylcholine, a key component of the plasma membrane. This way is also known as The Kennedy pathway [99]. Once phosphorylated, phosphocholine is trapped within the cell, which is crucial for PET imaging with ^11^C and ^18^F radiotracers based on choline. The second main pathway of choline metabolism is based on the oxidation of choline to betaine. It was first described by Ikuta and co-workers in 1977 [100] and involves the conversion of choline to betain by choline oxidase (E.C. 1.1.3.17) via a four-electron oxidation using two sequential FAD-dependent reactions [101]. However, the second pathway is not preferred for PET imaging applications using radiolabeled choline derivatives. To overcome this obstacle, ^11^C and ^18^F labeled choline derivatives, which are deuterated at the ethylene moiety and not in the immediate neighborhood of the desired radionuclide were applied due to their altered pharmacological behavior.

In 2003, Gadda investigated enzyme kinetics for choline oxidase with choline (**56**) and choline-D_4_ (**57**) as substrates to evaluate the impact of the kinetic isotope effect. It was shown that the oxidation of deuterated choline **57** was reduced to a minimum [101], which led to the successful development of choline-based radiotracers.

In 2009, Aboagye and colleagues compared the relative oxidation rates of the two isotopically radiolabeled choline species, [^18^F]fluorocholine ([^18^F]**58**) and [^18^F]fluorocholine-D_4_ ([^18^F]**59**) with respect to their metabolites [94]. Both betaine metabolites from [^18^F]**58** and [^18^F]**59** were obtained from mouse plasma after intravenous injection of both radiotracers. As a result, it was pointed out that [^18^F]**59** was remarkably more stable to oxidation than [^18^F]**58** with ~40% conversion of [^18^F]**59** to the betaine at 15 min after intravenous injection into mice compared to ~80% conversion of [^18^F]**58** to the respective betaine-metabolite.

In 2012, both ^11^C-labeled choline derivatives [^11^C]**56** and [^11^C]**57** as well as ^18^F-choline-D_4_ ([^18^F]**59**) were synthesized to compare their biodistribution and metabolic behavior. Additionally, the same group performed small-animal PET studies and kinetic analyses to evaluate the tracer uptake in human colon HCT116 xenograft-bearing mice [102]. It was found that the simple substitution of deuterium for hydrogen and the presence of ^18^F improves the stability and reduces degradation of the parent tracers. Furthermore, the availability is increased for phosphorylation and trapping within cells, which leads to a better signal-to-background contrast, thus improving tumor detection sensitivity of PET. In addition, deuterated ^11^C choline was demonstrated to have a higher stability compared to non-deuterated ^11^C-choline, but an increased rate of oxidation of betaine compared to ^18^F-D_4_-choline was observed. In 2014, the first promising human studies with healthy volunteers were accomplished [103].

### 4.1. Cycloalkyl Derivatives and Fluorine Connected to a Secondary Carbon Atom

**Scheme 16:**
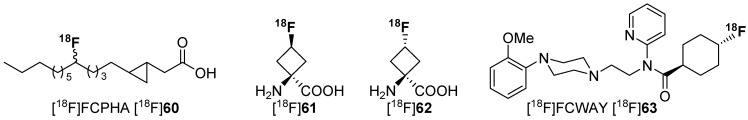
Selected examples of fluorine-18 bound to secondary carbon for stabilization.

Several literature sources reported that the replacement of an alkyl chain by a cycloalkyl ring resulted in more metabolically stable compounds [104,105,106,107]. Examples are given in Scheme 16. Despite this increased stability, only a few reports exist on PET radiotracers containing cycloalkyl rings. One example describes a potential radiotracer for assessing myocardial fatty acid metabolism, [^18^F]FCPHA [^18^F]**60**, containing a cyclopropyl moiety which allows the tracer to be trapped in the cells [108]. Another example describes non-natural ^18^F-amino acids with fluorine-18 located at the cycloalkyl residue [109,110]. [^18^F]**61** and [^18^F]**62** show an increased metabolic stability compared to their non-cyclic counterparts. The placement of ^18^F is especially important for compound [^18^F]**63**, because the methoxy group itself and also the introduction of a fluoroalkoxy moiety instead of the methoxy group at the phenol part of the molecule lead to a fast cleavage [111].

**Scheme 17:**
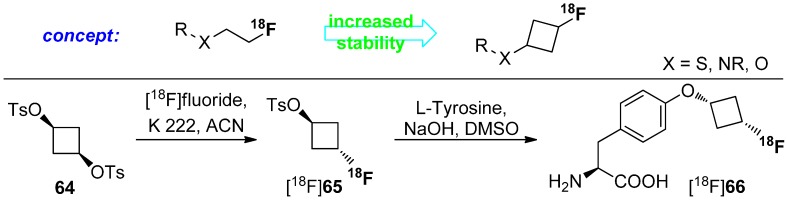
Labeling concept to avoid radiodefluorination and radiolabeling of L-tyrosine with [^18^F]fluorocyclobutyl tosylate ([^18^F]**65**).

Both non-natural amino acids were used as brain tumor imaging agent and W. Yu *et al*. [112] found that the newly developed amino acid [^18^F]**61** is comparable to [^18^F]**62**. However, the cyclic unnatural amino acids are not metabolized [113]. The major drawback of this approach is the stereoselective construction of the amino acid skeleton. Thus, Franck and colleagues reported a diverse approach using cyclic building blocks bearing the ^18^F-label. The research was focused on the metabolism of ^18^F-tracers with [^18^F]fluoroalkyl chains attached to hetereoatoms such as O, N, and S. Biotransformation (radiodefluorination) of these radiotracers was avoided by the utilization of cyclobutyl groups containing fluorine-18. Hence, cyclobutyl 1,3-ditosylate (**64**) was used as starting material. Radiofluorination was performed under standard conditions using [^18^F]F^−^, K 222 in anhydrous acetonitrile. After successful synthesis of the building block [^18^F]**65**, L-tyrosine was used and labeled. The concept and the full reaction path including radiolabeling are pointed out in Scheme 17.

The obtained [^18^F]fluorocyclobutyl derivative [^18^F]**66** is comparable with the well-known amino acid *O*-(2-[^18^F]fluoroethyl)-l-tyrosine ([^18^F]FET) in the case of cell uptake and blocking and showed an excellent metabolic stability in phosphate buffer and in human and rat plasma for 120 min [114,115].

Further, the connection of fluorine to a secondary carbon could also help to prevent radiodefluorination in some cases. However, when using ^18^F-FCWAY [^18^F]**63** (Scheme 16), the defluorination process is a major issue. To prevent degradation, the responsible enzyme (cytochrome P450 2E1 (CYP2E1) isozyme) is suppressed with miconazole nitrate prior to the injection of the radiotracer [116]. With this method it was possible to substantially avoid radiodefluorination and the combined uptake of [^18^F]fluoride in the skull.

### 4.2. SiFA-Techniology

The Si-F bond represents one of the strongest single bonds with a corresponding bond energy of 565 kJ/mol, which is 80 kJ/mol higher than the Si-C bond and suggest a high thermodynamically stability [117]. This fact led to the development of fluorine-18-radiotracers based on organosilanes, which should be unaffected against radiodefluoroination commonly associated with alkylfluorides. In 1985, Rosenthal and colleagues were the first who successfully radiolabeled [^18^F]fluorotrimethylsilane [118]. The reaction was performed using chlorotrimethylsilane as precursor with a yield of 65% and high radiochemical purity. However, subsequent *in vivo* investigation of [^18^F]fluorotrimethylsilane indicated a rapid hydrolysis followed by an enrichment of radioactivity in bones. For this reason, this concept was ineffective for the preparation of ^18^F-radiotracers [119,120].

**Scheme 18:**
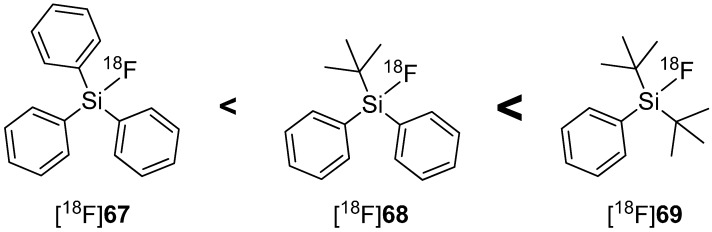
*In vitro* hydrolytic stability of [^18^F]fluorosilanes in dependence of their organic groups in human serum.

In 2000, Walsh and co-workers tried to induce the stabilization of Si-F-bond with bulky substituents such as phenyl or *tert*-butyl groups and confirmed the assumption of Rosenthal, who predicted the use of bulky substituents on silicon diminishes the hydrolysis of Si-F bond [121]. Furthermore, Choudhry and Blower investigated the behavior of different sized alkyl groups (Me, Ph, *tert*-Bu) and their combinations connected to fluorosilanes. The results showed that *tert-*butyldiphenyl[^18^F]fluorosilane ([^18^F]**68**) contained the highest stabilized Si-F-bond [122]. Contemporaneously, Schirrmacher and Jurkschat carried out comparable experiments and found di-*tert-*butylphenylfluorosilane ([^18^F]**69**) with the highest stability against hydrolysis of Si-F-bond and called this compound class SiFA (silicon-based fluoride-acceptor). The hydrolytic stability in dependence of the alkyl group is expressed in Scheme 18 [123].

**Scheme 19:**
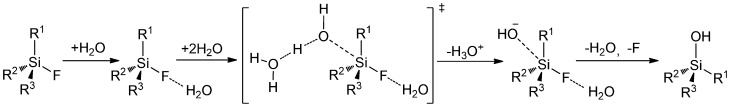
Suggested S_N_2 mechanism of the hydrolysis reaction of organofluorosilanes.

The high polarization of Si-F bond results in the kinetic instability of Si-F-bond [124] and allows an exchange under mild conditions. Due to the low energy of vacant d-orbitals tetravalent silicon as weak Lewis acid reacts with Lewis bases [125], which allows a nucleophilic attack by hydroxyl-groups in the case of aqueous conditions. Generally, nucleophilic displacement reactions on silicon proceed by the predicted S_N_2 mechanism in Scheme 19 [126,127].

Contrary to carbon, a real pentagonal transition state including hypervalent silicon is formed and assists this substitution. The larger covalent radius of silicon compared to carbon contributes to this nucleophilic substitution [128], which led to the poor kinetic stability of Si-F-bond despite the high thermodynamic stability. Thus, a stabilization of Si-F bond to prevent a nucleophilic attack is only possible by raising the sterical bulkiness of the substituents. This fact explains the weak impact (plain structure) of phenyl moieties and they are also responsible for the augmented Lewis acid properties of silanes.

**Scheme 20:**
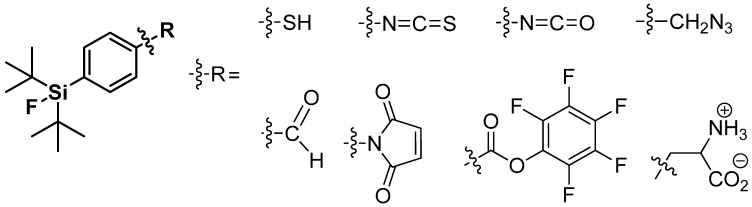
A summary of applied SiFA building blocks taken from the review by Bernard-Gauthier *et al*., 2014 [128].

Only the use of *tert-*butyl groups located in direct neighborhood of Si-F-bond prevents hydrolysis due to their bulky three-dimensional structure. The third substituent on the silicon is utilized for further derivatization. Hence, the phenyl group seems to be the perfect choice for a functionalization with groups such as aldehydes, NCS-, or -SH in mainly para-position to the silyl residue. These resulting building blocks were often used for labeling of peptides and proteins [123,129,130,131]. An overview is given in Scheme 20. Furthermore, the use of alkyl-groups as third substituent with supplemental functionalization was proven, but exhibited a reduced hydrolytic stability compared to the phenyl tracers [126,132].

**Scheme 21:**
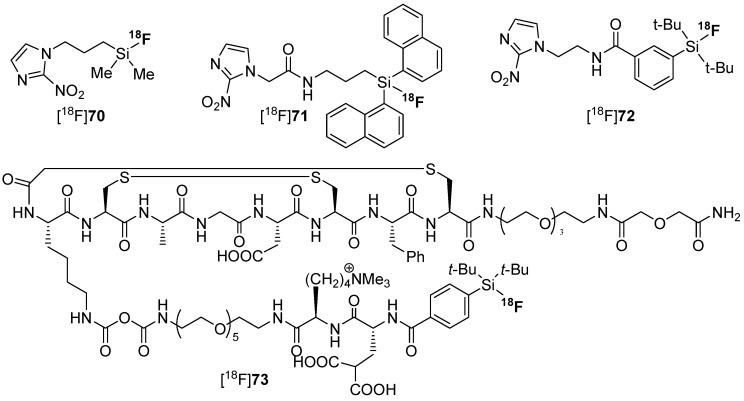
Different hypoxia tracers [^18^F]**70**–[^18^F]**72** with diverging metabolic stability [135] and an example for biomacromalecule [^18^F]**73** labeled with SiFa [131].

Next, the introduction of fluorine-18 was evaluated by the use of different leaving groups such as alkoxycarbonyl-groups [122,133], by halogen and isotope exchange [118,123] and by applying hydrosilanes [134]. Manifold examples for the use of the SiFA concept were octreotide, bombesin, RGD, PSMA, antibodies, simple molecules, carbohydrates, and biotin. An excellent overview is provided by an outstanding review, see [128]. In most of the cases, the respective building blocks were applied especially for the biomacromolecules, but also a direct introduction of fluorine-18 was accomplished. However, the direct introduction exhibited a rather low yield compared to the building block approaches. Examples of hypoxia tracers [^18^F]**70**–[^18^F]**72** with rising metabolic stability and [^18^F]**73** as SiFA-labeled peptide is found in Scheme 21.

## 5. Miscellaneous

### 5.1. Fluorosulfonamides

**Scheme 22:**
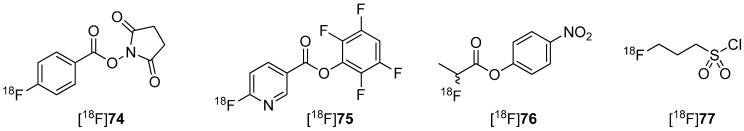
Selected examples for ^18^F building blocks for radiolabeling of peptides.

Metabolically stable building blocks also referred to as prosthetic groups were required especially for the radiolabeling of peptides or other biomacromolecules. Conventional building blocks such as [^18^F]SFB [^18^F]**74** were used to radiolabel particularly with primary amine residues of peptides (N-terminus or lysine) under formation of amide (peptide) bonds. A selection of these ^18^F-building blocks is pointed out in Scheme 22.

**Scheme 23:**
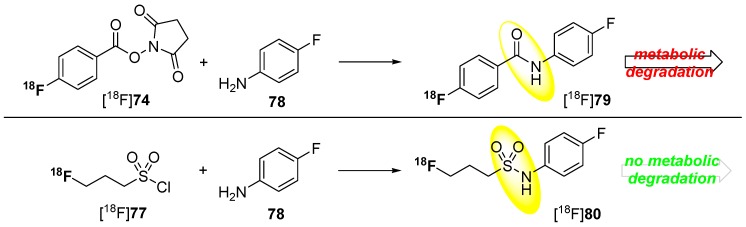
Comparison in radiofluorination and metabolic stability for [^18^F]SFB [^18^F]**74** and [^18^F]fluoropropylsulfonyl chloride [^18^F]**77**.

However, this kind of radiofluorinated aromatic fluoroacetamides turned out to be unstable *in vivo* and undergoes *N*-defluoroacylation [136]. It was reported that this degradation may be caused by the involvement of carboxylesterase (E.C. 3.1.1.1) or other hydrolases [137,138]. As an alternative to these acyl-based prosthetic groups, the 3-[^18^F]fluoropropanesulfonyl chloride ([^18^F]**77**) was introduced by Li *et al*. [139] and by Löser and co-workers [140]. They substantiate the metabolic integrity of fluorinated sulfonamide: *N*-(4-fluorophenyl)-3-fluoropropane-1-sulfonamide (**80**) compared to the aromatic acyl derivative *N*-(4-fluorophenyl)-fluoroacetamide (**79**) in a spectrophotometric enzyme assay using pig liver esterase. Both compounds are shown in Scheme 23.

After 120 min (approx. one half-life of ^18^F), only 20% of the starting ^19^F-compound **79** was intact whereas, at the same time point, over 95% of the sulfonamide **80** was still detectable. Furthermore, pseudo-first order kinetics for the degradation of the acylamide could be determined.

### 5.2. Click-Chemistry

**Scheme 24:**
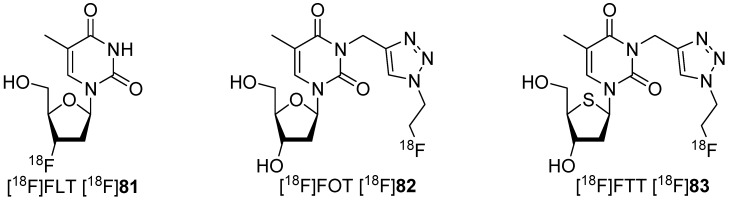
Presentation of ^18^F-labeled thymidine derivatives in the case of their labeling position.

A further approach to avoid degradation was using triazoles [141], which were obtained by click chemistry [142,143]. Two different research groups investigated [^18^F]fluoroalkyl groups bound at position N-3 of the triazole moiety of thymidine derivatives like [^18^F]**82** with conflicting results and uncertainty over the metabolic stability of the radiotracers *in vivo*. The prepared ^18^F-tracers are shown in Scheme 24. Smith and colleagues postulated that 1,4-disubstituted triazoles have a higher metabolic stability *in vivo* due to the greater steric bulk of the triazole. The metabolic stability is increased relative to simple fluoroalkyl substituents to thymidine-phosphorylase-mediated cleavage [144,145].

Initial studies of the ability of these nucleosides to undergo phosphorylation demonstrated that [^18^F]FLT [^18^F]**81** was phosphorylated to approx. 7%–8% after 60 min incubation, whereas no phosphorylation was observed with [^18^F]FOT [^18^F]**82** over the same time period. Comparison with [^18^F]FLT [^18^F]**81** showed that [^18^F]FOT [^18^F]**82** was poorly phosphorylated at the 5-position of the deoxyribose residue. The poor thymidine kinase 1 (TK1) substrate tolerance due to substitution at nitrogen N-3 was given as a possible reason for this finding.

**Scheme 25:**
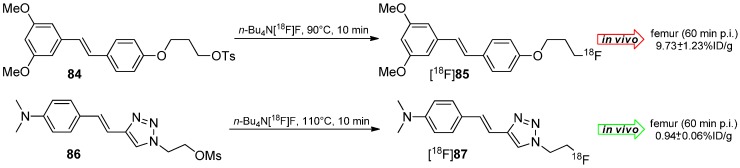
Radiolabeling and selected *in vivo* results of reservatrol [^18^F]**85** and styryltriazole [^18^F]**87**.

The working group of Choe developed ^18^F-Labeled styryltriazole and resveratrol derivatives such as [^18^F]**85** and [^18^F]**87** for β-amyloid plaque imaging [146]. Compounds **84** and **86** were labeled under standard labeling conditions (*n*-Bu_4_N[^18^F]F, acetonitrile, 90 °C or 110 °C, 10 min) and yielded both tracers in 20%–30% RCY for [^18^F]**85** and 56% RCY for [^18^F]**87** with a A_S_ ≈ 38 GBq/µmol and a RCP > 99% (Scheme 25).

*In vivo* studies of both tracers showed a remarkable metabolic degradation of reservatrol derivative [^18^F]**85** under elimination of [^18^F]fluoride which was accumulated in the femur (16.15% ± 3.10% ID/g after 120 min). Conversely, the styryltriazole compound [^18^F]**87** showed almost no cleavage of [^18^F]fluoride (1.54% ± 0.02% ID/g after 120 min).

### 5.3. CF_3_-Derivatives

As stated in the introduction (Section 1.1), the use of CF_3_ groups could increase the metabolic stability of pharmacologically relevant compounds and radiotracers [147] due to the increased bond strength of the C-F bond in this group compared to single fluorine connected to carbon and due to the higher steric shielding of the carbon center. Furthermore, the trifluoromethyl group is present in a large number of agrochemicals, biologically active drugs and anesthetics, which led to attempts to introduce fluorine-18 to yield [^18^F]CF_3_ group containing radiotracers; see an excellent review by Lien and Riss [148].

Normally, the introduction of [^18^F]fluoride was accomplished via ^18^F/^19^F isotopic exchange [149,150,151], Lewis acid mediated reactions [152,153], halogen for ^18^F exchange [154,155,156] or H^18^F addition [157] and electrophilic reactions with [^18/19^F]F_2_ [158,159], but most of these reactions suffer from low specific activities due to the carrier added reactions and/or rough conditions.

**Scheme 26:**
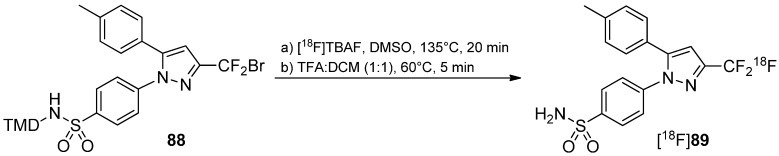
Radiolabeling of [^18^F]CF_3_ containing Celecoxib derivative [^18^F]**89**.

An example is presented regarding the synthesis of 4-[5-(4-methylphenyl)-3-([^18^F]trifluoromethyl)-1*H*-pyrazol-1-yl]benzenesulfonamide ([^18^F]Celecoxib) ([^18^F]**89**) which is known to be a selective COX-2 inhibitor [160]. The labeling procedure was accomplished exchanging bromide with [^18^F]F^−^ using [^18^F]TBAF in DMSO at 135 °C (Scheme 26). [^18^F]Celecoxib was achieved in 10% ± 2% RCY (end of synthesis) with >99% chemical and radiochemical purity and a specific activity, which was 4.40 ± 1.48 GBq/μmol (end of bombardment). *In vitro* stability experiments showed only a small amount of [^18^F]fluoride coming from radiodefluorination in 10% ethanol-saline (6.5% after 4 h). However, *in vivo* experiments of [^18^F]**89** with Wistar rats showed a higher skeleton uptake compared to brain or heart; regions where COX-2 is known to be present due to the radiodefluorination process. In contrast, no uptake in skull and skeleton was observed in baboon indicating only a low degree of defluorination of [^18^F]**89** *in vivo*. In addition, metabolite analyses show that [^18^F]**89** undergoes fast metabolism. Polar metabolites were found in baboon plasma and 17.0% of unmetabolized tracer was determined at 60 min after injection; no evidence was obtained for free [^18^F]fluoride.

### 5.4. ^18^F-Fluoroborates

An impressive stability was found for the B-F bond (645 kJ/mol) in BF_3_ [117]. Thus, the introduction of fluorine-18 directly connected to boron represents a further promising alternative to avoid radiodefluorination. The non-binding electrons of fluorine atoms in BF_3_ form π-bonds with boron, which represent partial double bonds with an average bond length of 130 pm. Based on this fact, the still electron demanding boron center is less hydrolytic unstable. The previously sp^2^-hybridized boron center is changed to sp^3^ by accepting an electron pair of an additional fluoride in the former p_z_-orbital to form a tetrafluoroborate anion (BF_4_^−^). Thereupon, the bonds in BF_4_^−^ are single bonds with also a high hydrolytic stability [117,161]. 

This basic principle is used for the creation of fluourine-18-containing boron derivatives. Exchange of F^−^ in these species is rare due to the aforementioned high bond strength of the B-F bond. The fluorine atoms in BF_4_^−^ are substitutable but the exchange should be advisedly chosen. In general, the exchange of fluorine by other halogens leads to weaker bonds [162]. Calculations for triarylfluoroborates predicted a weakening of remaining B-F bond [163]. Therefore, comparable functionalizations will be necessary to apply this concept for the development of radiotracers.

**Scheme 27:**
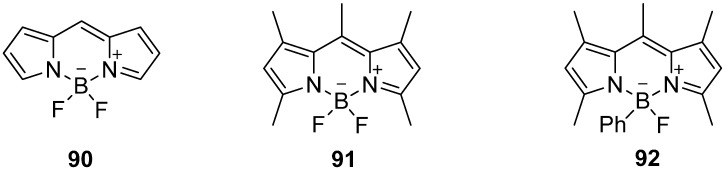
Core structure **90** of selected bodipy derivatives **91** and **92**.

The application of bodipy derivatives represents a promising approach. Several methylated compounds such as **91** and **92** were described for the first time by Treibs and Kreuzer in 1968 in addition with the excellent fluorescence properties of these dyes [164]. However, the synthesis of the core structure **90** succeeded first in 2009 [165,166,167], see Scheme 27.

**Scheme 28:**
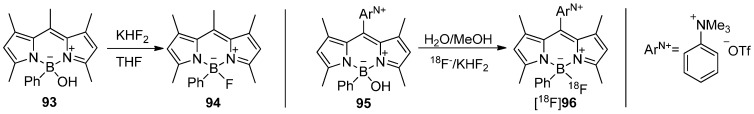
Conversion of OH compound **93** to **94** and radiolabeling of **95** to yield [^18^F]**96**.

In 2011, bodipy dyes moved into focus for radiolabeling with fluorine-18. For that purpose, a radiolabeling building block based on a modified bodipy was created by Li and co-workers [168]. The radiosynthesis of the BPh[^18^F]F core containing derivative [^18^F]**96** was simply realized by using KHF_2_/[^18^F]F^−^ (directly from target water without drying) in water/methanol starting from BPhOH-precursor 95 with a A_S_ = 0.9 GBq/µmol. The solubility of the precursor **95** and the desired bodipy derivative [^18^F]**96** was achieved due to the ammonium triflate moiety ArN^+^. The results are summarized in Scheme 28.

**Scheme 29:**
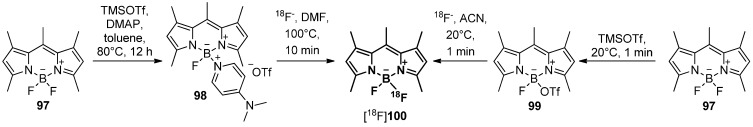
Preparation of the building block [^18^F]**100** from compound **97** via either precursor **98** or **99** under mild labeling conditions.

In 2012, an alternative radiolabeling building block based on the B[^18^F]F_2_ core containing bodipy derivative was prepared [169]. Thus, the respective precursors were either synthesized via an exchange of one of the fluorides of **97** by a DMAP leaving group using TMS triflate/DMAP to yield **98** or directly by changing one fluoride to triflate with TMS triflate to yield **99**. The desired DMAP/^18^F exchange of **98** to [^18^F]**100** succeed but the triflate precursor **99** was proven to be more effective due to the higher radiochemical yields of [^18^F]**100**, milder reaction conditions and a short reaction time during the labeling reaction (Scheme 29). A labeling building block based on [^18^F]**100** was further used for the successful labeling of Trastuzumab with fluorine-18.

Both groups demonstrated the high metabolic stability of the desired bodipy derivatives *in vitro* as well as *in vivo*. No radiodefluorination in terms of an accumulation of activity in the skeleton due to free [^18^F]fluoride was observed. Based on these results, this concept has great potential to create stable radiotracers having a B-[^18^F]F bond.

## 6. Conclusions

Radiodefluorination is one of the most important metabolic degradation processes for ^18^F-radiotracers due to the release of [^18^F]fluoride *in vivo*, which is then accumulated in the skull and bones. This undesired accumulation leads to PET images that are false-positive in terms of skeleton imaging or comprise a bad signal to background ratio.

Several efforts have been made in the past to avoid this defluorination or to considerably reduce it. The insertion of deuterium to stabilize the C-F bond seems to be the most successful approach. Thus, a building block strategy was developed using small deuterated molecules with ^18^F-label. In many cases, radiodefluorination could be reduced in an appreciable manner.

Other approaches can show reduced radiodefluorination in a remarkable manner as well. The introduction of deuterium in other positions relative to ^18^F (or ^11^C) is also promising. In this case, the stabilization is used to reduce cleavage of other parts of the molecule. Furthermore, the introduction of C[^18^F]F_3_ groups leads to a reduced degradation since the bond strength of the C-F bond is increased in the CF_3_ group. Finally, the insertion of a [^18^F]fluorocyclobutyl moiety is favored over open fluoroalkyl chains due to the increased steric demand and, therefore, reduced metabolism. The same effect can be reached by the utilization of special functional groups to avoid cleavage on this position.

Binding [^18^F]fluorine to heteroatoms like silicon or boron offers also the possibility to obtain radiotracers, which show reduced radiodefluorination. Though, in the case of silicon based ^18^F-radiotracers, the additional protection of the Si-[^18^F]F center with bulky substituents is mandatory. The use of [^18^F]bodipy derivatives offers the chance to use the same molecule for PET as for optical imaging.
